# A ten-year (2009–2018) database of cancer mortality rates in Italy

**DOI:** 10.1038/s41597-022-01729-0

**Published:** 2022-10-21

**Authors:** Roberto Cazzolla Gatti, Arianna Di Paola, Alfonso Monaco, Alena Velichevskaya, Nicola Amoroso, Roberto Bellotti

**Affiliations:** 1 Department of Biological, Geological and Environmental Sciences (BiGeA), Alma Mater Studiorum – University of Bologna, Bologna, Italy; 2 Institute for BioEconomy (IBE), National Research Council of Italy (CNR), Rome, Italy; 3 Dipartimento Interateneo di Fisica, Università degli Studi di Bari Aldo Moro, Bari, Italy; 4 Istituto Nazionale di Fisica Nucleare, Sez. di Bari, Italy; 5 Biological Institute, Tomsk State University, Tomsk, Russia; 6 Dipartimento di Farmacia – Scienze del Farmaco, Università degli Studi di Bari Aldo Moro, Bari, Italy

**Keywords:** Cancer, Cancer

## Abstract

In Italy, approximately 400.000 new cases of malignant tumors are recorded every year. The average of annual deaths caused by tumors, according to the Italian Cancer Registers, is about 3.5 deaths and about 2.5 per 1,000 men and women respectively, for a total of about 3 deaths every 1,000 people. Long-term (at least a decade) and spatially detailed data (up to the municipality scale) are neither easily accessible nor fully available for public consultation by the citizens, scientists, research groups, and associations. Therefore, here we present a ten-year (2009–2018) database on cancer mortality rates (in the form of Standardized Mortality Ratios, SMR) for 23 cancer macro-types in Italy on municipal, provincial, and regional scales. We aim to make easily accessible a comprehensive, ready-to-use, and openly accessible source of data on the most updated status of cancer mortality in Italy for local and national stakeholders, researchers, and policymakers and to provide researchers with ready-to-use data to perform specific studies.

## Abstract (summary)

**Table:** 

Measurement(s)	Standardized Mortality Ratio
Technology Type(s)	Public database

## Background & Summary

The prevailing theory, formulated around the 1950s, considers cancer as a set of about 200 diseases characterized by abnormal cell growth, escaping the normal control mechanisms of the organism^1^. The process of transformation of a normal cell into a neoplastic cell occurs through various stages with the accumulation of genetic, functional, and morphological anomalies^2^.

The most known causes of DNA alterations in the genesis of cancer include environmental pollution, genetic alteration, infections, and unhealthy lifestyles such as tobacco and alcohol over-consumption^3,4^. However, in some cases, no specific causes can be still attributed to neoplastic cell formations, In Italy, approximately 400,000 new cases of malignant tumors are recorded every year, of which 200,000 in men and 180,000 in women. Overall, every day, about 1,000 Italian citizens receive a new malignant cancer diagnosis^5^.

Excluding skin cancers (non-melanoma), prostate cancer prevails in men which accounts for ~20% of all diagnosed cancers; follow by the tumor of the lung (15%), colorectal (14%), bladder (12%), and stomach cancer (4%). Breast cancer accounts for ~30% of women’s cancers, followed by colorectal (12%), lung (12%), thyroid (5%), and uterus (5%)^6^.

As in many other industrialized areas, in Italy cancers are the second cause of death (~30% of all deaths), after cardiovascular diseases (37%). In men, cancers and cardio-circulatory diseases cause approximately the same number of deaths (~35%) while in women the cardio-circulatory diseases are more relevant than tumors (40% vs 25%)^5^. Therefore, the probability of dying from cancer in Italy is approximately 1 out of 3 for men and 1 out of 4 for women^5^.

The frequency of deaths caused by tumors in the Italian areas covered by the Cancer Registers is, on an annual average, about 3.5 deaths per 1,000 men and about 2.5 per 1,000 women, for a total of about 3 deaths every 1,000 people^7^. These data, if scaled on a daily average, suggest that every day about 500 people die in Italy because of a tumor. Nevertheless, during the last 40 years, Italians’ life expectancy increased by about 10 years in both sexes^8^. Moreover, if in the 1950s the Italian population was mainly made up of children and very few elderly people, in 2050 the forecast is a population consisting largely of elderly people and few children. This entails an increased risk of developing cancer in a population older and affected by comorbidities^8^.

Furthermore, there exists a heterogeneous distribution in Italy for the main epidemiological indicators of cancer (incidence, survival, mortality, and prevalence), with a North-South gradient for most tumor types^7^. In fact, the standardized incidence rate (in the European population) for all cancers among men is ~5% lower in the Center and ~15% in the South and Islands compared to North and for women by ~5% and ~17%, respectively. Underlying these differences may be protective factors (different lifestyles, food habits, reproductive factors) that persist in the regions of Central and South/Islands, but mainly a lower exposure to carcinogenic factors (in particular, environmental pollution; see for a recent report^9^).

Overall, survival has recently increased by 54% in 2005–2009 against 51% in 2000–2004, 46% in 1995–1999, and 39% in 1990–1994 for men and by 63% against 60%, 58%, and 55% in the corresponding periods for women^10^. Specifically, there is an improvement in survival for some of the most frequent tumor sites: colorectal (currently 65% for both sexes), female breast (87%), and prostate (91%). However, for some poor prognosis cancers, survival improvements have been limited in recent years, as in the case of lung cancer, pancreas, and gallbladder^7^.

Nonetheless, it is worth noting that, the global cancer mortality rate has not always and not everywhere significantly decreased^11^, despite almost a century of advanced research to find cures for tumors and improve survival.

For instance, during the last years, in Italy, mortality has decreased significantly in the whole country except in the South and Islands, where the rates are substantially unchanged^7^.

**Fig. 1:**
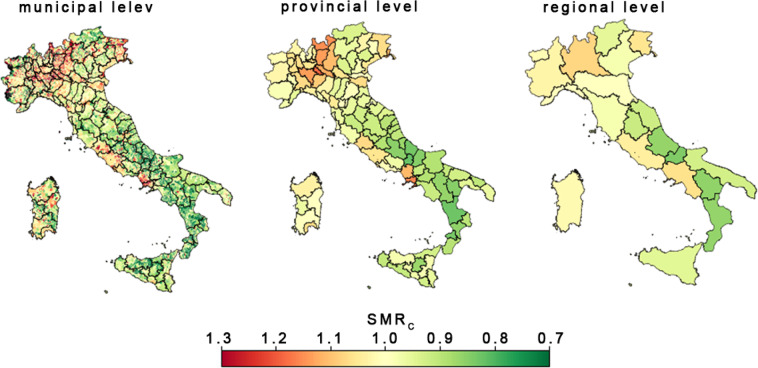
Geographical distribution of average standardized mortality rate for malignant tumors (SMR_C_) at three different administrative levels: municipal, provincial and regional.

Although several indicators and reports are developed every year to monitor the cancer situation in Italy, more recent, long-term (at least a decade) and spatially detailed data (up to the municipality scale) are neither easily accessible nor available for public consultation by the citizens, scientists, research groups and associations. Here we present a ten-year (2009–2018) database on cancer mortality rates (in the form of Standardized Mortality Ratios; hereafter SMR) for all macro-types of cancers in Italy at municipal, provincial, and regional scales (Fig. 1). This dataset aims to make available a comprehensive, ready-to-use, and openly accessible source of data on the most updated status of cancer mortality in Italy for local and national stakeholders, researchers, and policymakers and to provide researchers with ready-to-use data to perform specific studies.

The SMR dataset^12^ is available on the Dryad public data repository for open access. Source data, supplementary information, and Python codes to build the dataset are available on Zenodo^13,14^.

## Methods

### Data source

The list of variables required for building the SMR database is reported in Table 1. The study period encompasses ten years: 2009–2018 and 23 macro-categories of cancer types listed in Table 2. Raw data, except for the observed number of deaths by cause at the municipal level, were retrieved from the public data warehouse of the Italian National Institute of Statistics (ISTAT, http://www.istat.it/en/, last access: 26/01/2022), a public organization producing the official statistics in Italy. The observed number of deaths by cause at the municipal level was shared by the ISTAT upon request.

**Table 1:** List of symbols, definitions, and data used to compute the SMR for different cancer types and territorial levels of aggregation.

Symbol	Definition	Source
*Provinces/Municipality/regional level*		
*SMR*	Standardized Mortality Ratio	Calculated from Eq. (1)
*°m*	observed number of deaths by cause	ISTAT, personal communication
^*E*^*m*	number of expected deaths by cause	Calculated from Eq. (2)
*n*_*i*_	age-specific census data on population	ISTAT, data warehouse
*Country level* *(i.e., Reference variable)*		
^*M*^*R*_*i*_	age-specific death rates by cause of reference population	Calculated from Eq. (3)
M_i_	age-specific number of deaths by cause of reference population	ISTAT, data warehouse
N_i_	age-specific census data on the reference population	ISTAT, data warehouse

Capital letters are used for reference population variables whereas lower-case letters are for local variables (i.e. municipal, provincial and regional ones). Variables with the subscript “*i*” are age-specific.

**Table 2:** IDC-10 denominations and codes.

Tumor type (European Shortlist)	IDC-10 code	IDC-10 Full code	ID in the SMR DATABASE
***malignant tumors***	***C00-C979***		***C***
“of which malignant tumors of the lips, oral cavity and pharynx”	C00-C14	C000-C149	1
“of which malignant tumors of the esophagus”	C15	C150-159	2
“of which malignant stomach tumors”	C16.1-C17	C161-C179	3
“of which malignant tumors of the colon, rectum, and anus”	C18-C21	C180-C219	4
“of which malignant tumors of the liver and intrahepatic bile ducts”,	C22	C220-C229	5
“of which malignant tumors of the pancreas”	C25	C250-259	6
“of which malignant tumors of the larynx”	C32	C320-329	7
“of which malignant tumors of the trachea, bronchi, and lungs”	C33-C34	C330-C349	8
“of which skin malignant melanoma”	C43	C430-C439	9
“of which malignant breast tumors”	C50	C500-509	10
“of which cervical malignant tumors”,	C53	C530-539	11
“of which malignant tumors of other parts of the uterus”	C54-C55	C540-C559	12
“of which malignant tumors of the ovary”	C56	C560-569	13
“of which malignant prostate tumors”	C61	C610-619	14
“of which malignant kidney tumors”	C64	C640-649	15
“of which malignant bladder tumors”	C67	C670-679	16
“of which malignant tumors of the brain and central nervous system”	C70-C72	C700-C729	17
“of which malignant thyroid tumors”	C73	C730-739	18
“of which Hodgkin’s disease and lymphomas”	C81-C85	C810-C859	19
“of which leukemia”	C91-C95	C910-C959	20
“of which other malignant tumors of lymphatic/hematopoietic tissue”	C86-C90; C96	C860-909; C960-969	21
of which other malignant tumors	Remaining codes between C000-C979		22
*non-malignant tumors (benign and of uncertain behavior)*	*D0-D489*	*D000-D489*	*D*

Details on single variables are provided in the following subparagraphs. Source data supporting the computation of the SMR for different cancer types and levels of aggregation were uploaded to the SMR Database.

### Age-specific number of deaths by cause of reference population (*M*_*i*_)

ISTAT provides the deaths by age and causes occurring in Italy by aggregating information from the attending physician, registrars, and necropsies, with annual updates. *M*_*i*_ is available on the I.Stat data warehouse (http://dati.istat.it/?lang=en, last access 22/11/2021) following the path: *Health statistics, Causes of death, Cause and age*. From an interactive window on the I.Stat portal it is possible to customize the request of data, selecting the territory (from provinces to national level), the age or age-group (arranged by 5 years), gender, causes of death, and year. To our purposes, we selected the total number of deaths at national level by causes listed in Table 2 grouped into 20 age-groups of 5 years each whose intervals are: 0–4, 5–9, 10–14, 15–19, 20–24, 25–29, 30–34, 35–39, 40–44, 45–49, 50–54, 55–59, 60–64, 65–69, 70–74, 75–79, 80–84, 85–89, 90–94, over 95 years.

### The age-specific census data on population at municipal (*n*_*i*_) and national level (*N*_*i*_)

ISTAT estimates the resident population by age based on population censuses that occurred in 2018, 2011, and 2001. The last update of the census data was released in March 2021. The total number of resident population at the municipal level was retrieved on the I.Stat data warehouse following the path: *Population and Households, Inter Censuses Population, Estimated Resident Population for years 2002*–2019. The population size at the upper levels (i.e., provincial, regional, and national) was determined by aggregation.

At the time this document was written, Italy counted 7093 municipalities distributed over 110 provinces. However, since the number of municipalities and provinces had changed frequently in the last decades, the number of existing municipalities may change over the years. Indeed, some municipalities no longer exist (since absorbed by other municipalities), while others changed denominations. Moreover, few municipalities have missing data. The estimates of annual SMR considered all these variations (see “Data Processing”).

### Deaths on the resident population by cause (°m)

Data on mortality at the municipal level are available only upon request to the ISTAT cont@ct center (https://contact.istat.it/index.php?Lingua=Inglese, last access:22/11/2021). For privacy reasons, data on mortality at the municipal level omit the age distributions (from personal communication). Moreover, for the same reason, if the frequency of deaths by municipality and sex is less than 3, ISTAT obscures the cause of death; Furthermore, in a few cases, the municipality might not be indicated due to errors in the death form compiled by doctors or for a lack of understanding.

Data on mortality provided by ISTAT are encoded according to the International Classification of Diseases and Related Health Problems (ICD-10), an international disease classification system defined by the *World Health Organization (WHO)*^15^ and includes all the causes of death as reported in^16^. Table 2 provides the IDC-10 definitions and codes for cancer’s causes of death used for the computation of the Italian SMR Database.

### Ancillary datasets

Several ancillary datasets were also included in the analysis: *i*) the list of statistical codes and denominations of administrative units (i.e. municipalities, provinces, and regions) adopted by the ISTAT (available at https://www.istat.it/it/archivio/6789, last access: 20/10/2021, last release 2019); *ii*) the list of statistical codes and denominations of municipalities that have been abolished since 2009. Such a list provides both the old statistical codes and denominations of the abolished municipalities and the current ones; *iii*) The shapefile of administrative units available at https://www.istat.it/it/archivio/222527, last access 22/11/2021) to map the results at municipal- and regional-level scale.

## Data Processing

### Computation of SMR

As most causes of death vary significantly with people’s age and sex, data on mortality are commonly analyzed through a standardized index to improve comparability over time and between areas.

A versatile index for neutralizing the effects of age structure is the Standardized Mortality Ratio (SMR)^17,18^. The SMR expresses the real differences in disease frequency of a study cohort compared to the general population (i.e. *Reference Population*).

Henceforth, the whole Italian population would be used as the reference population and capital letters will be used for all the reference variables, namely the reference population size and mortality, whereas lowercase letters will refer to variables at the local scale (i.e. municipal, provincial or regional scale). For example, the variables expressing the number of deaths for the reference and municipal population are “*M*” and “*m*”, respectively. Moreover, since the SMR is a weighted average of the age-specific mortality rates (see below), where each weight accounts for the ratio of people within an age group compared to the reference population, a subscript “*i*” is used to indicate a given age-group, where *i* = 1,2, …, I with I = 20 being the number of the age-groups of 5 years each.

The procedure presented here to estimate the SMR can be used for either municipal, provincial or regional levels. The estimation of Italian SMR at municipal and provincial levels represents a novel ready-to-use database, while that for the regional level is used for the technical validation.

For a given locality, year, and cause of death, the SMR is the ratio between the observed number of deaths (°*m*) and the number of expected deaths (^E^*m*):

1 $$SMR=\frac{{}^{O}m}{{}^{E}m}$$

Where *°m* should be an available observational data and ^E^*m* is estimated as the weighted sum of age-specific population size for the given locality (*n*_*i*_) per age-specific death rates of the reference population (^*M*^*R*_*i*_):

2 $${}^{E}m={\sum }_{i=1}^{I}{}^{M}{R}_{i}\times {n}_{i}$$

^*M*^*R*_*i*_ could be provided by a public health organization or be estimated as the ratio between the age-specific number of deaths of the reference population (*M*_*i*_) to the age-specific reference population size (*N*_*i*_):

3 $${}^{M}{R}_{i}={M}_{i}\times {N}_{i}$$

Thus, the value of ^*E*^*m* is weighted by the age distribution of deaths and population size.

SMR assumes value 1 when the number of observed and expected deaths are equal. Hence, if the incidence of a given cause of death was equally distributed over the entire reference population, the score of SMR for a given locality and year would approximate 1.

In real life, the SMR is commonly different from 1 since the incidence of a given cause of death could be strongly affected by some local environmental and/or socio-economic factor. For a given locality and/or year, the more the value of SMR is greater than 1, the more the mortality incidence compared to the expected one (i.e. *excess of deaths*), while the more the value of SMR is lower than 1, the lower is the mortality incidence (i.e. *defect of deaths*). Therefore, for any given locality showing an excess of deaths beyond those expected (i.e., SMR >1), there must be another one with a defect of expected deaths (i.e., SMR <1). Overall, the distribution of SMR across the whole reference population is centered around 1.

Following Eqs. (1–3), the SMR was computed for each year of the period 2009–2018 and for a single cause of death listed in Table 2 by using the data listed in Table 1 at three different levels of aggregation: municipal, provincial (equivalent to the European classification NUTS 3) and regional (i.e., NUTS2). The SMR was also computed for the broad category of malignant tumors (i.e. C00-C979, hereinafter cancer macro-type C), and the broad category of malignant tumors plus non-malignant ones (i.e. C00-C979 plus D0-D489, hereinafter cancer macro-type CD).

At the time of writing this paper, Italy counts 7093 municipalities. Hence, to aid data comparison both on a spatial and temporal scale, the SMR of single years refers to the currently existing 7093 municipalities whose list of denominations and codes (from ISTAT) are also included in the SMR Database. Data on both mortality and population size from no longer existing municipalities were aggregated (summed) into the municipality to which they currently belong.

The same reasoning applied to the provinces and regions: the SMR at the upper levels of aggregations was estimated for the current provinces (107 units) and regions (20 units) by aggregating municipal data on mortality (*°m*) and population size by age (*n*_*i*_).

Along with the SMR values for single years, we added the time-series average and related 90% and 95% lower confidence levels (when at least three years of real value exist) as additional ready-to-be-used data. Indeed, many epidemiologic studies suggest adopting cautionary lower confidence levels for statistical elaboration and/or descriptive statistics of SMR^19–21^.

A lower 90% and 95% confidence interval were computed according to the Byar method^17^. However, since the SMR Database provides the SMR values for single years, a user can decide to calculate the confidence limits differently or with different confidence thresholds.

According to the Byar method, the approximate lower (*α*_*low*_) and upper (*α*_*up*_) limits for a specified confidence level (*α*), are:

4 $${\alpha }_{low}{=}^{O}m{\left(1-\frac{1}{{9}^{O}m}-\frac{{z}_{\alpha /2}}{{3}^{O}{m}^{1/2}}\right)}^{3}$$

and

5 $${\alpha }_{up}=({}^{O}m+1){\left(1-\frac{1}{9({}^{O}m+1<?RemoveMO1?>)}+\frac{{Z}_{\alpha /2}}{3{({}^{O}m+1)}^{1/2}}\right)}^{3}$$

with *z*_*α*/2_ is equivalent to the 100(1-α) percentile of the standard normal distribution N(0,1), with (1-α) equal to the desired confidence level. Accordingly, Z = 1.64 and 1.96 for 90% and 95% confidence level, respectively.

Knowing the lower limit for a 100(1-α)% confidence level, the lower confidence level of SMR is given by

6 $$SM{R}_{low}=\frac{{\alpha }_{low}}{{}^{E}m}$$

The resulting SMRs are presented on the outline of a relational database where the municipal statistical code works as a key feature. Data are presented as a Comma Separated Value file (CSV) of 7904 rows (i.e. 7093 municipalities) per 14 columns (i.e., administrative statistical code, SMR for single years embracing the period 2009–2018, mean, and lower 90% and 95% confidence levels).

### Missing data and exceptions

Before computing the SMR, two major issues were addressed: missing data and available data from abolished municipalities. These issues were both present in mortality data at the municipal level. We assumed that a municipality should record at least one death per year for whatever cause among those included in the data source. If such a record exists, even for causes other than cancer, then the data on causes listed in Table 2 is considered present yet equal to zero (i.e., zero cancer deaths). Conversely, if a municipality does not have any mortality records for a given year, then data for such a municipality and year is considered missing.

Data on deaths from abolished municipalities, when available, had been used for the computation of the yearly SMR. To this end, data on deaths from abolished municipalities were aggregated to the current belonging municipality through a sum. Overall, municipalities with missing data on deaths range between 4.4% (in 2009) and 5.5% (in 2014) compared to the total of currently existing 7903 municipalities. The number of abolished municipalities has progressively decreased over the study period from 313 in 2009 to 79 in 2018.

The age-specific census data on population had only 6 municipalities with missing data and the list of municipalities embraces only those currently present. This means that the aggregation of census data from abolished municipalities has already been done by the ISTAT.

Overall, when for a given year a municipality has missing data on mortality or resident population, the SMR value is not calculated and marked as “nan” (i.e., not-a-number).

Census and deaths data on reference populations had no missing value. Missing data for a given municipality and year return a missing value on the SMR for that municipality, year, and all the cancer types.

## Data Record

The ten-year (2009–2018) database of Italian cancer mortality rates is available for download on Dryad^12^. Specifically, the database contains the SMR data for the period 2009–2018 by cause listed in Table 2 at three levels of aggregation: municipal, provincial and regional; On Zenodo^13,14^ are also available *ii*) the figure maps of average SMR for single cancer types and levels of aggregation; *iii*) the scripts in Python language to reproduce the elaboration along with the raw source data.

Within the root folder in Dryad (“DATA”) there are three main sub-folders: “SMR”, “Observed mortality”, and “Expected mortality”. The first one holds data on SMR, while the second and third ones hold data on the observed and expected number of deaths (i.e., ^O^m and ^E^m), respectively.

Data are provided for each level of aggregation (i.e., municipal, provincial, and regional) in specific sub-folders, hence in each of the three main folders, there are other three sub-folder. The sub-folders in “SMR”, in turn, contain the computation of ten-year SMR by cause as listed in Table 2 plus that for CD for single administrative units in Comma Separated Values (CSV) files. The format of CSV files is always the same across the levels of aggregation: in the rows are the single administrative units (i.e. municipalities or provinces or regions); the columns report the statistical code of the administrative units (first column), the value of SMR for the years 2009–2018 (2th-11th columns), the ten-years average (12th column), the 90% and 95% lower confidence levels (13-14th column) estimated according to the Byars method. A “readme.txt” file is present in each SMR sub-folders to easily access and understand the data. An example of the SMR records in a CSV file is given in Table 3.

**Table 3:** Example of SMR data presented in a CSV file (cause C, municipal level, available in the dataset under the path “SMR\SMR_municipal_level\SMRc.txt”.

Admin code	2009	2010	2011	2012	2013	2014	2015	2016	2017	2018	Average SMR	SMR low c.l.
1001	1.7297	1.0801	0.8701	0.9874	1.0577	1.0782	1.4161	1.1176	1.6081	1.6928	1.2638	1.0735
1002	0.7838	1.0394	0.638	1.5703	0.977	1.4358	0.802	0.4499	1.3033	1.4421	1.0442	0.8724
1003	1.5996	nan	nan	0	0.7427	0.7479	nan	1.4279	0.7144	0.7162	0.8498	0.4182

Missing data are tracked as not-a-number (nan).

Similarly, within the main folder of ref. N2 (“Figure maps”, provided as additional information) on Zenodo there are three sub-folders, one for each level of aggregation, holding the maps of average SMR for single cancer type as listed in Table 2. Lastly, in the main folder of ref. N3 (“scripts”, provided as additional information) there are the source data used for the computation of SMR, the scripts used to produce all the elaborations, the shapefiles of administrative boundaries, and some intermediate outputs saved as pickle file, namely a homologous Python module extent (“pkl”) that can be reloaded to produce figures and further analysis in the python language by end-users. The Python script files are denoted through leading ordinal numbers that reflect the order of execution to reproduce the results.

## Technical Validation

To ascertain that the estimation of SMR was as accurate as possible, two checks were made. First, since the SMR refers to a reference population, data on mortality at the municipal and national levels must be consistent. This means that the sum of deaths by cause for a given year among municipalities should be equal to the national number of deaths for the same cause and year. However, due to a few cases where data are omitted or not available, the total deaths by cause and year at the municipal level can be fewer than those at the national one. The higher the difference, the more the resulting SMR is biased. Moreover, such a proof of consistency allows us to also confirm that the procedure and the encoding for the selection of specific IDC-10 causes were properly implemented.

Table 4 shows the differences between deaths provided at the national level and those obtained from the sum of deaths by year and cause at the municipal level. Values in Table 4 are expressed as percentages in relation to the national score. In most cases, the differences are less than 0.5% with the only exception for death causes C16.1-C17 (i.e., malignant stomach tumors) where the differences range between 1–2%. Such low percentages of discrepancies between the national and municipal data are completely consistent with what is reported by ISTAT (i.e. few cases where municipalities are omitted or cause of deaths blacked out) and widely acceptable in terms of SMR precision which would suffer a small underestimation (0–2%).

**Table 4:** Difference between deaths at the national level and those obtained from the sum of municipalities by year and cause of death.

Tumor type (IDC-10)	2009	2010	2011	2012	2013	2014	2015	2016	2017	2018
*C00-C979*	0.41	0.40	0.40	0.39	0.41	0.45	0.35	0.33	0.31	0.34
*D0-D489*	0.36	0.32	0.27	0.27	0.30	0.32	0.15	0.37	0.26	0.28
*C00-C979 Plus D0-D489*	0.44	0.43	0.40	0.39	0.41	0.43	0.33	0.37	0.34	0.34
C00-C14	0.60	0.44	0.46	0.25	0.46	0.72	0.28	0.34	0.54	0.37
C15	0.84	0.43	0.22	0.44	0.44	0.44	0.38	0.62	0.78	0.27
C16.1-C17	1.13	1.18	1.41	1.51	2.02	2.01	1.46	1.01	1.01	1.36
C18-C21	0.27	0.35	0.36	0.38	0.33	0.35	0.26	0.33	0.22	0.29
C22	0.48	0.29	0.36	0.33	0.35	0.44	0.24	0.48	0.27	0.39
C25	0.41	0.43	0.41	0.28	0.33	0.41	0.36	0.25	0.25	0.29
C32	0.29	0.41	0.49	0.32	0.45	0.60	0.34	0.37	0.31	0.26
C33-C34	0.33	0.29	0.29	0.28	0.36	0.27	0.27	0.25	0.24	0.26
C43	0.34	0.28	0.33	0.16	0.26	0.40	0.36	0.30	0.15	0.44
C50	0.31	0.37	0.30	0.31	0.36	0.29	0.21	0.21	0.25	0.20
C53	1.04	0.25	0.46	0.73	0.00	0.44	0.00	0.79	0.62	0.20
C54-C55	0.44	0.25	0.36	0.25	0.28	0.40	0.24	0.15	0.45	0.53
C56	0.45	0.31	0.44	0.31	0.33	0.32	0.25	0.34	0.36	0.27
C61	0.43	0.33	0.24	0.33	0.31	0.43	0.46	0.35	0.25	0.31
C64	0.37	0.48	0.43	0.52	0.29	0.33	0.26	0.32	0.30	0.26
C67	0.40	0.39	0.30	0.26	0.23	0.32	0.25	0.19	0.35	0.27
C70-C72	0.26	0.32	0.19	0.40	0.33	0.43	0.34	0.29	0.34	0.21
C73	0.49	0.37	0.18	0.34	0.18	0.38	0.36	0.38	0.00	0.18
C81-C85	0.40	0.49	0.32	0.30	0.28	0.25	0.27	0.40	0.62	0.62
C91-C95	0.24	0.38	0.42	0.31	0.23	0.30	0.22	0.23	0.24	0.27
C86-C90; C96	0.25	0.28	0.21	0.09	0.23	0.20	0.31	0.08	−0.39	−0.39
Remaining codes in between C000-C979	0.03	0.10	−0.08	−0.14	−0.29	−0.23	−0.19	0.11	0.05	−0.18

Consistency check. Percentage in relation to the deaths at national value.

The second check consisted of the comparison of the SMR with the Standardized Mortality Rates provided by the ISTAT (SMR_istat) at the regional level. Such an index contains information very similar to the SMR here described, albeit different in absolute terms since it expresses the standardized rate, namely the number of deaths per 100,000 inhabitants compared to a reference population, rather than a ratio as in our case (Eq. 1).

Hence, the values of SMR at the regional level must be strongly correlated to the SMR_ISTAT albeit much different in absolute values.

**Fig. 2:**
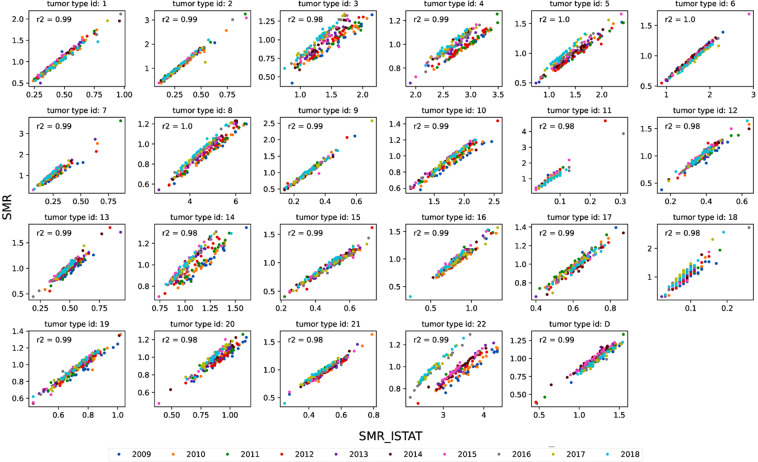
SMR_ISTAT vs. SMR scatter plot for single causes listed in Table (2). Correlation coefficients as the average of correlation coefficients between SMR and SMR_ISTAT for single years.

Figure 2 shows the scatter plots between SMR and SRM_ISTAT and related coefficients of Pearson correlation by causes listed in Table 2. The correlation between the two indices is very strong in all the cancer types (R^2^ > 0.91). It is worth noting that from Fig. 2 some discrepancies between years in the SMR_istat emerged, for instance, in the cancer type “of which other malignant tumors “ after 2016. Specifically, the SMR_ISTAT data in Fig. 2 seems divided into two broad clusters, one embracing the years 2016–2018, and one embracing 2009–2015. Indeed, starting from the reference year 2016, ISTAT has adopted the edition of the ICD-10 updated to 2016 for the codification of the causes of death which presents numerous changes in the guidelines for the selection of the initial cause, as well as some modifications in the classification of some pathologies compared to the previous one used by ISTAT up to 2015 (i.e. ICD-10 2009 version, further information available at https://www.istat.it/it/archivio/6708, last access 22/11/2021). The tumor type “of which other malignant tumors”, embraces all the tumor types other than those specified in Table 2 and it could be more sensitive to the variations of the IDC-10 coding system.

## Usage Notes

**Fig. 3:**
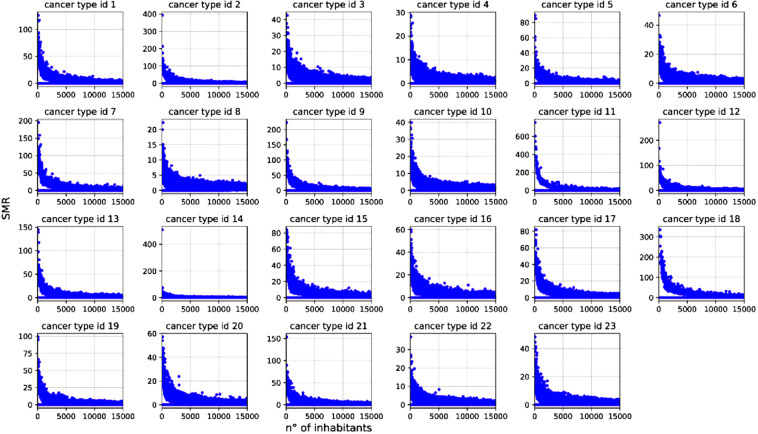
Scatter plot of SMR (at the provincial level, all years) vs. the number of inhabitants for single causes listed in Table 2. In small municipalities, the computation of SMR in a given year could show extremely high values (see “Usage notes”). The use of average SMR and related lower confidence levels allow overcoming possible large inter-annual variability in small populations.

The interannual variability of SMR for a given administrative unit might be large under small populations. Indeed, being the SMR a rate standardized over the population size, the expected mortality (i.e., ^*E*^*m*) in small populations might result low (e.g. 10^−2^) and in turn, according to Eq. 1, even a few deaths (say 1 or 2) in a year could yield a relatively high SMR as shown in Fig. 3. For this reason, we recommend avoiding using single-year estimates and using the SMR at lower 90% or 95% confidence intervals averaged over 3–5 years, at least.

## Data Availability

Data used for the production of the Italian SMR database are available from ISTAT (see the paragraph *Data Source*). The elaborations have followed the procedure described in the Methods section and can be implemented in whatever numerical computing environment (e.g., R, Matlab, Python). In our case, the algorithms used were created in Python 3.7 and are available on Zenodo^14^.
